# CTA Imaging of Peripheral Arterial Injuries

**DOI:** 10.3390/diagnostics14131356

**Published:** 2024-06-26

**Authors:** Stefania Tamburrini, Giulia Lassandro, Francesco Tiralongo, Francesca Iacobellis, Francesco Michele Ronza, Carlo Liguori, Rosita Comune, Filomena Pezzullo, Michele Galluzzo, Salvatore Masala, Vincenza Granata, Antonio Basile, Mariano Scaglione

**Affiliations:** 1 Department of Radiology, Ospedale del Mare, ASL NA1 Centro, 80147 Naples, Italy; 2 Radiology Unit 1, University Hospital Policlinico “G. Rodolico-San Marco”, 95123 Catania, Italy; 3 Department of General and Emergency Radiology, “Antonio Cardarelli” Hospital, 80131 Naples, Italy; 4 Department of Diagnostic Imaging, AORN S. Anna and S. Sebastiano, 81100 Caserta, Italy; 5 Division of Radiology, Università degli Studi della Campania Luigi Vanvitelli, 80138 Naples, Italy; 6 Department of Emergency Radiology, San Camillo Forlanini Hospital, 00152 Rome, Italy; mgalluzzo@sirm.org; 7 Department of Medicine, Surgery and Pharmacy, University of Sassari, 07100 Sassari, Italy; 8 Division of Radiology, Istituto Nazionale Tumori IRCCS Fondazione Pascale-IRCCS di Napoli, 80131 Naples, Italy; 9 Department of Medical and Surgical Sciences and Advanced Technologies “GF Ingrassia”, University Hospital Policlinico “G. Rodolico-San Marco”, 95123 Catania, Italy

**Keywords:** peripheral arterial injuries, CTA, CT angiography, vascular trauma, blunt trauma, penetrating trauma, dissection, transection, pseudoaneurysm

## Abstract

Traumatic vascular injuries consist of direct or indirect damage to arteries and/or veins and account for 3% of all traumatic injuries. Typical consequences are hemorrhage and ischemia. Vascular injuries of the extremities can occur isolated or in association with major trauma and other organ injuries. They account for 1–2% of patients admitted to emergency departments and for approximately 50% of all arterial injuries. Lower extremities are more frequently injured than upper ones in the adult population. The outcome of vascular injuries is strictly correlated to the environment and the time background. Treatment can be challenging, notably in polytrauma because of the dilemma of which injury should be prioritized, and treatment delay can cause disability or even death, especially for limb vascular injury. Our purposes are to discuss the role of computed tomography angiography (CTA) in the diagnosis of vascular trauma and its optimized protocol to achieve a definitive diagnosis and to assess the radiological signs of vascular injuries and the possible pitfalls.

## 1. Introduction

Vascular trauma accounts for 3% of all traumatic injuries [1,2,3]. Vascular injuries of extremities can occur isolated or in association with major trauma and other organ injuries, and they account for 1–2% of patients admitted to the emergency room (ER) and account for approximately 50% of all arterial injuries [4,5,6], with a prevalence of lower extremities injuries over that of upper extremities in the adult population [7]. 

Traumatic vascular injury is characterized by damage to arteries and/or veins. Typical consequences are hemorrhage and ischemia, and the outcome of vascular injuries is strictly correlated to the environment and the time background [8]. 

Treatment delay can cause disability or even death, especially for limb vascular injury. Physiological and preclinical studies have revealed that muscle damage is present at 3 h of ischemia and is near complete and irreversible at 6 h [9,10]. Vascular peripheral injury is highly morbid, and limb salvage is a rule rather than an exception [11,12]; it can occur in isolation or as a part of polytrauma, and the treatment can be challenging because of the dilemma of which injury should be prioritized [11,12].

We aim to discuss the role of computed tomography angiography (CTA) in diagnosing vascular trauma and its optimized protocol to achieve the definitive diagnosis and to assess the radiological findings of vascular injuries and the possible pitfalls.

### 1.1. Types of Peripheral Vascular Injuries

One of the most important things to understand is that vascular injury is not synonymous with active bleeding. 

Vascular injury manifestations include hemorrhage and ischemia. Vessel injuries can manifest as vasospasm, contusion, intimal flaps, intimal disruption or hematoma, external compression, laceration, transection, focal wall defect with pseudoaneurysm, and arteriovenous fistula [13,14]. Vessel disruption is the most common injury and can be complete or incomplete. It may also present as an expanding or pulsatile hematoma. 

Complete disruption presents with active hemorrhage decreasing in time due to spasm and thrombosis. In incomplete disruption, blood flow can be maintained, ischemia may not occur, and a pseudoaneurysm can develop [15]. Intimal injuries lead to thrombosis or intimal flap formation that may cause distal ischemia. 

Dissection caused by intimal injuries may also have a delayed presentation. In penetrating traumas, arteriovenous fistulas may frequently occur in relation to the narrow course between arteries and veins. Furthermore, these lesions may have a delayed presentation. Arterial spasm determines reversible limb ischemia, but all other vascular lesions should be first excluded [15,16].

Limb ischemia is the primary cause of concern in distal extremity injuries with peripheral arterial trauma, whereas proximal transections of the axillo-subclavian or ilio-femoral axis represent a life-threatening risk of exsanguination due to the greater caliber of the vessels and the greater difficulty of compression in these locations [5]. 

### 1.2. Causes of Peripheral Vascular Injuries

**Figure 1:**
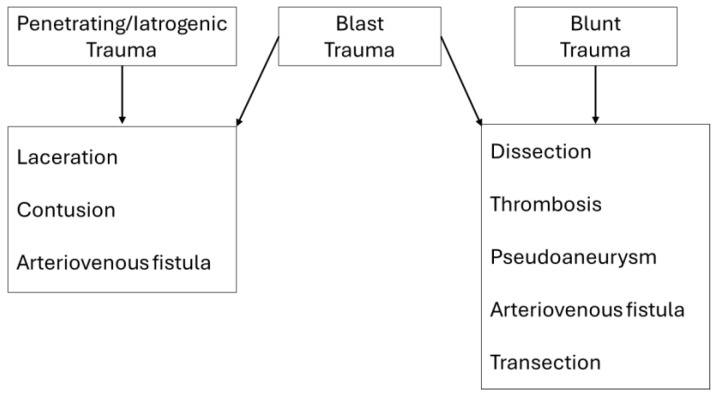
Causes of Peripheral Vascular Injuries.

Vascular injury can occur in blunt, penetrating, blast, and iatrogenic trauma (Figure 1). In civilian settings, blunt trauma has always been the leading cause of injury, but penetrating trauma is becoming more frequent because of the increase of urban violence [17,18,19,20]. 

Gunshot injuries depend on projectile velocity, mass, and characteristics [21,22], and they determine severe tissue damage. Penetrating stab wounds have low-energy character and constitute 70–90% of cases [23] and usually result in lacerations or transection without contusion [24]. 

Vessel transection can be complete or incomplete; in complete transection, the artery often retracts and spasms, with sequential thrombosis; instead, massive bleeding can occur in incomplete transection. In penetrating trauma, according to the extent of the wound, various clinical manifestations may occur, from a puncture wound with minimal bleeding and minute signs of peripheral ischemia to a large laceration of the skin with life-threatening hemorrhage [25]. 

Blunt vascular trauma is the effect of the shearing or compressive forces that lead to contusion, tearing, and dissection of the blood vessel, and it is often the result of major soft-tissue loss and concomitant fractures [14,26]. Blast trauma is the consequence of explosive detonation, and a duplex mechanism may damage the vessel. The blast wave determines the shrapnel secondary fragments that damage vessels directly, as occurs in penetrating trauma; on the other hand, the blast wind determines body displacement and injury, which are related to the blunt mechanism [15,16]. 

Iatrogenic vascular injuries are defined as injuries sustained to an artery during an operation and percutaneous interventions, and they are becoming more frequent with the development of mini-invasive and endovascular techniques [27,28]. 

The most common complication of vascular access in the endovascular approach is hematoma and pseudoaneurysm in the access site [29]. The rate of these incidents varies between 0.5 and 1.0% and recently was decreased using various vascular sealing systems. When arterial vessels are iatrogenically damaged, massive bleeding is rare. Arteriovenous fistula, intimal lesion, thrombosis, rupture, acute limb ischemia, and pseudoaneurysm may also occur. The common femoral artery is the most common site of this trauma, and damage may occur at the vessel access point, at the site of intervention, or anywhere in between [15]. 

Vascular injury during orthopedic surgery is not a frequent complication, with an incidence of 0.05–0.1% [30,31]. During hip or knee replacement, the mechanism of injury is usually indirect, as in blunt trauma, from torsion and elongation forces that result in intimal tear and vessel thrombosis. During open repositions, the mechanism is usually direct, as in penetrating trauma, caused by a stabilization material and fixation that results in arterial damage. The common injury sites are in the upper limbs, including the armpit, the medial part of the arm, and the ulnar fossa, due to a superficial position of the vascular structures, and in the lower limbs, including the groin, the medial thigh area, and the popliteal fossa [25]. Most peripheral vascular injuries involve the superficial femoral artery or brachial artery [32]. Vascular trauma may or may not be associated with bone fractures, and the absence of bone fractures cannot exclude the presence of vascular damage. Common fractures associated with loss of distal perfusion are those with bone displacement, segmentation, comminution, or floating joint [5]. The most common location is the femur with an associated wedge-shaped or butterfly fragment at a level close to Hunter’s canal, fracture dislocations of the knee, and fracture dislocations of the ankle. Severe soft-tissue and bony injury distal to the trifurcation of the leg arteries are frequently associated with injury to all three calf vessels with often non-viable muscle in multiple compartments and segmental bone loss with a high risk of amputation.

### 1.3. Primary Survey 

Specific procedures of bleeding control should be carried out during the pre-hospital settings. Open extremity fractures occur in an environment of high energy transfer [33] and are rarely associated with major hemorrhage. Control of catastrophic hemorrhage is the first stage of the primary survey, together with airway management and cervical spine stabilization (CABCDE: Circulation (exsanguinating hemorrhage), Airway, Breathing, Circulation, Disability, and Exposure) [34]. 

Hemorrhage control may be achieved by applying direct wound compression and compression dressing to the source of major bleeding and contemporaneously maintaining tissue perfusion. 

Tourniquets have been used in military settings, although in civilian settings, the use of limb-constrictive devices before the onset of hemorrhagic shock provides temporary control of hemorrhage and precious time to transfer the patient safely to the hospital [15]. A tourniquet is usually applied on the arm or thigh and less often in distal areas (forearm, below the knee), usually 8 cm above the suspected vascular lesion [25,33]. 

The time of tourniquet application must be noted or written to avoid unnecessarily prolonged ischemia. Blind clamping of an actively bleeding vessel is potentially detrimental to vascular tissue, and the accompanying nerves and should be avoided. 

Hemostatic dressings and substances are used, and they usually take 3–5 min to initiate coagulation between the dressing and injury site [35,36]. Whether accompanied by major hemorrhage or not, a devascularized limb associated with an open fracture [37] is a clinical emergency requiring prompt recognition and treatment. 

**Figure 2:**
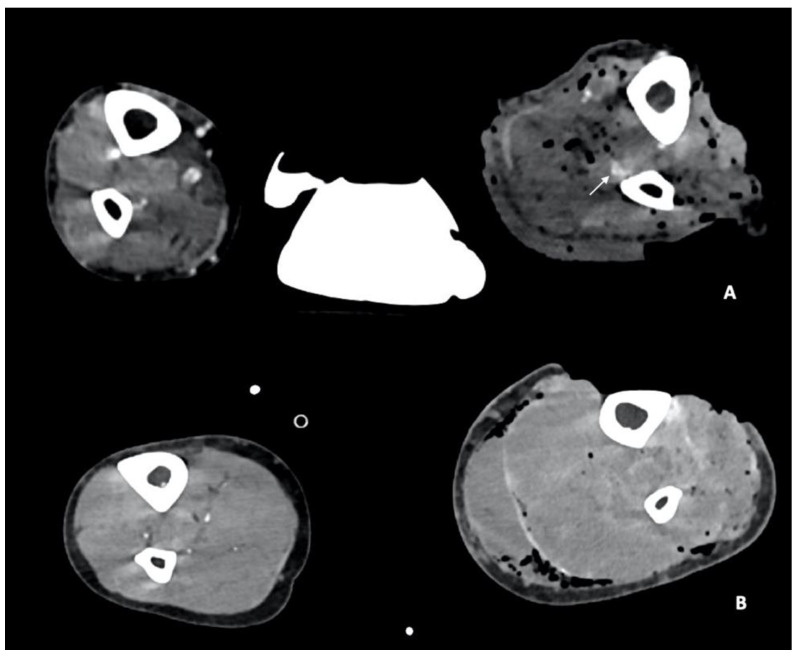
CTA, axial planes (**A**,**B**). According to Gustilo–Anderson classification, these two different patients were classified as grade IIIB (**A**) and IIIC (**B**), respectively, involving at least three of the four major systems: integument, soft tissue, bone, and nerves and vessels. In both these patients, the left lower-limb fractures are characterized by extensive bone loss, periosteal stripping with devitalized fragments, massive contamination, and poor soft-tissue coverage. In (**A**), the left peroneal artery contusion can be noted (arrow), while in (**B**), the left arteries cannot be recognized, indicating arterial injuries that require reperfusion.

In this setting, the Gustilo–Anderson classification, a grading system of open fractures, is used to predict limb-threatening septic complications that require secondary amputation based on the degree of soft-tissue injury in mangled extremity injuries. These injuries (type IIIB and IIIC) involve at least three of the four major systems: integument, soft tissue, bone, and nerves and vessels [38] (Figure 2). Type IIIC fractures are characterized by extensive bone loss, periosteal stripping with devitalized fragments, massive contamination, poor soft-tissue coverage, and arterial injuries that require reperfusion [5]. Approximately 40% of Gustilo type III fractures are associated with significant arterial injuries [5,37,39,40]. Blood loss and hemodynamic instability are higher in proximal artery injuries, and upper- and lower-limb arterial injuries may present differently because of morphological differences in vessel size and muscle compartments [7]. Recognition is based on hard clinical signs, including lack of palpable pulses, continued bleeding, or an expanding hematoma [41]. 

Fractures or joint dislocations should be reduced, as this may restore distal circulation. The assessment of the pulse should not rely on the use of Doppler ultrasound and the ankle–brachial pressure index (ABPI) [42].

## 2. Diagnosis of Peripheral Vascular Injuries

### 2.1. Clinical Manifestations

Hemorrhage and tissue ischemia are the main clinical manifestations of vascular trauma. Symptoms of lower-limb vascular injuries can be described as hard or soft signs. Hard signs include arterial bleeding, loss of pulse, expanding hematoma, bruit or thrill, and signs of ischemia, and these indicate the need for immediate surgical intervention [5]. The classic 6P syndromes, defined as paresthesia, pulselessness, paralysis, pain, pallor, and poikilothermia, can diagnose damage to lower-limb arteries. Soft signs include a history of prehospital blood loss, diminished pulse, moderate hematoma, proximity to a large vessel or bony injury, and ipsilateral neurologic deficit, and these indicate the need for further diagnostic imaging [11,12,43]. It is important to underline that negative clinical exams do not rule out vascular trauma, especially in the calf, where low blood compensation can masquerade the vessel injury [11,12,44].

### 2.2. Auxiliary Examination

The ankle–brachial index (ABI) is the ratio of ankle blood pressure to brachial blood pressure and can identify limb ischemia. A normal ABI index (>0.90) has a high sensitivity to rule out vascular injury in the lower limb; instead, an ABI < 0.90 necessitates further investigation [45,46]. ABI represents an important diagnostic tool in evaluating lower-limb injuries [10,11,44], but preexisting peripheral vascular disease makes it less reliable, necessitating additional imaging [11,12]. Many traumatic vascular lesions can be occult on clinical examination, such as non-flow-limiting pseudoaneurysm of inline arteries and transections of non-line arteries such as the profunda femoris [47].

### 2.3. Imaging

Ultrasound (US) is widely used in the setting of trauma, and peripheral vascular examination may detect features of vascular injuries such as luminal narrowing, intramural hematoma, flaps, posttraumatic stenosis, the “yin-yang” sign characteristic of pseudoaneurysm, and acute occlusion [11,12,48,49]. 

The use of color Doppler increases ultrasound accuracy and has a sensitivity up to 94% [11,12,50]. US has several limits in the diagnosis of peripheral vascular injuries with a certain false-negative rate. 

Ultrasound is operator-dependent and requires experienced staff, and more importantly, it cannot access some areas due to bony structures, open wounds, large hematomas, bulky dressing, or splints [11,12,51]. Moreover, high BMI and subcutaneous emphysema negatively impact ultrasound examination [11,12,52]. In penetrating trauma, ultrasound is not sensitive enough to rule out vascular injuries [52,53,54]. 

Generally, Doppler US and the ankle–brachial pressure index (ABPI) are associated with a substantial false-negative rate and inter-observer variability [15,33,42], so they are not routinely used to rule out vascular injuries; instead, positive US may obviate CTA [52,55].

Despite digital subtraction angiography (DSA) being considered the gold standard in peripheral vascular injuries, allowing diagnosis and treatment, the technological progress of multidetector computed tomography (MDCT) scanners makes MDCT with CT angiography (CTA) the imaging of choice in evaluating patients with suspected peripheral artery injuries, replacing DSA as the first diagnostic step and allowing an accurate definition of peripheral vascular injuries and other associated trauma lesions [11,12]. 

Moreover, because vascular limb traumas can occur in isolation or as a part of polytrauma, a total body CTA may be performed all at one time, allowing the detection of vascular limbs and other cranial, neck, thoracic, abdominal, pelvic, and musculoskeletal injuries. Currently, 85% of patients with multi-system trauma undergo whole-body trauma CT [10,56] on admission, with simultaneous considerations of extremities and intracavitary injuries [32,41,57]. 

CT angiography is not indicated routinely in polytraumatized patients but is used in the case of risk factors (open fractures, distal tibia fractures, multiple fractures in one extremity, or isolated fractures of the proximal third of the fibula) along with at least one of hard or soft signs (hard signs: absent distal pulses, pulsatile bleeding, cold/pale limb, expanding hematoma, palpable thrill, and audible bruit; soft signs: decreased pulses compared to the contralateral side, any peripheral nerve deficit(s), small local hemorrhage(s), a wound near an artery, and non-pulsatile hematoma) [5,30,38,55,56,58]. 

However, in patients with suspected vascular injuries, a negative CTA is also used as rationale for immediate discharge [59].

## 3. MDCT or CTA

### 3.1. CTA Protocol

A specific protocol for a patient with suspected limb trauma should be chosen both on the parameters of the scanner and whether the CTA is acquired alone or as part of a whole-body examination (Table 1) [5].

The patient is supine-positioned feet first. The scanning width and position of the limbs depend on the context: In polytrauma patients, the volume is extended to the lower limbs, with legs at the isocenter to the gantry and feet slightly externally rotated; containment bands are always preferred if injuries allow them and especially in uncooperative patients. The upper limbs involved are imaged in adduction along the flanks, favoring the traumatized side in centering the patient; depending on the size of the patient, one or both upper limbs can be included in this way. 

In the case of isolated limb trauma, the position of the lower limbs is identical, while the positioning of the upper limbs depends on the type of trauma. If possible, the injured upper limb is placed over the head with the palm raised and fingers extended; if not, the arm is scanned in a prone position with the upper limb adducted along the flanks. In some severely injured upper-limb trauma, the patient may be not able to mobilize the arm, and it can be scanned adducted to the body [57,60]. 

All devices that can generate artifacts, such as rings and chains, should be removed before scanning if possible. Pillows and tape can be used to immobilize the upper extremity and fingers as much as possible. Concerning the contrast agent, the higher the iodine concentration, the better the quality of the study due to the higher density of the vessels. Vascular venous access is obtained with an angiocath caliber of 18 or 20 gauge, which adequate for the flow (at least 3 mL/s), followed by a 40 mL saline flush at the same rate. 

The positioning of the intravenous cannula should be chosen concerning the body area to be studied; for evaluating the lower limbs, the venous access should be positioned in the antecubital fossa and on the opposite side of the injured arm to prevent the dense venous contrast obscuring the arterial side. 

However, in the case of a study of both upper limbs, in the absence of a central venous access, a peripheral venous access could be chosen, which, however, would not allow the use of high flows [61]. The amount of contrast agent depends on the patient’s weight, iodine delivery rate (IDR), and on the length of the scanning duration. The examination starts with a biplane scout topogram to prescribe the scanning range and FOV. 

CT scans are acquired in the caudal–cranial direction from the inferior aspect of the aortic arch to the tips of the fingers and in the cranio–caudal direction when the upper limbs are placed above the head [60]. It is suggested to acquire an unenhanced scan to focus spontaneous hyper densities such as bone fragments and compare with post-contrast acquisitions to better understand the contrast agent distribution. 

If a dual-energy CT machine is available, the use of a virtual unenhanced scan can be considered as well as iodine maps that may help detect vascular alterations [62]. 

Then, a multiphasic CT study is suggested with arterial portal phase with delayed phase acquisition in selected cases [63]. The arterial scanning delay is determined by automated bolus tracking with the region of interest on the aortic arch for the upper-limb examinations or in whole-body CT examination and on the abdominal aorta in case of lower-limb examinations. Automated bolus tracking is recommended for optimal acquisition timing, particularly in patients with decreased cardiac output. 

The venous scan is acquired about 60–70 s after the contrast agent injection and is essential to detect venous injuries and bleeding and to differentiate contained vascular injuries from actively bleeding lesions [64]. 

The late phase, acquired 180 s after the contrast agent injection, offers further help in detecting late bleeding and solving doubts [64]. 

Multiplanar reconstruction, maximum intensity projection (MIP) reconstruction, and volume-rendered (VR) and CTA road maps are extremely useful in the assessment of limbs’ vascular trauma [5,44,57,58] and need to be routinely adopted in the postprocessing [60].

### 3.2. CTA Imaging Findings

**Figure 3:**
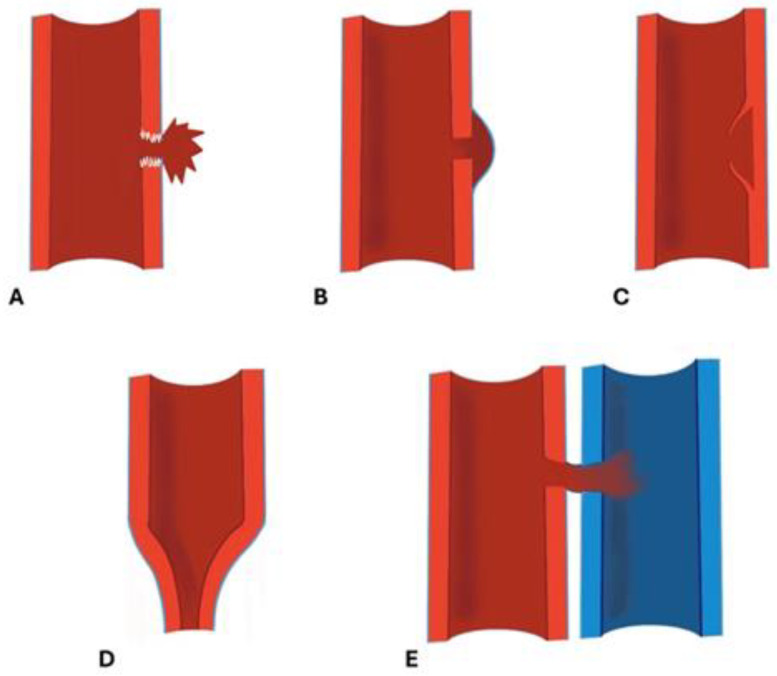
The drawing shows the main findings of arterial trauma, represented by arterial transection (**A**), pseudoaneurysm (**B**), dissection (**C**), luminal narrowing (**D**), and arteriovenous fistula (**E**).

**Figure 4:**
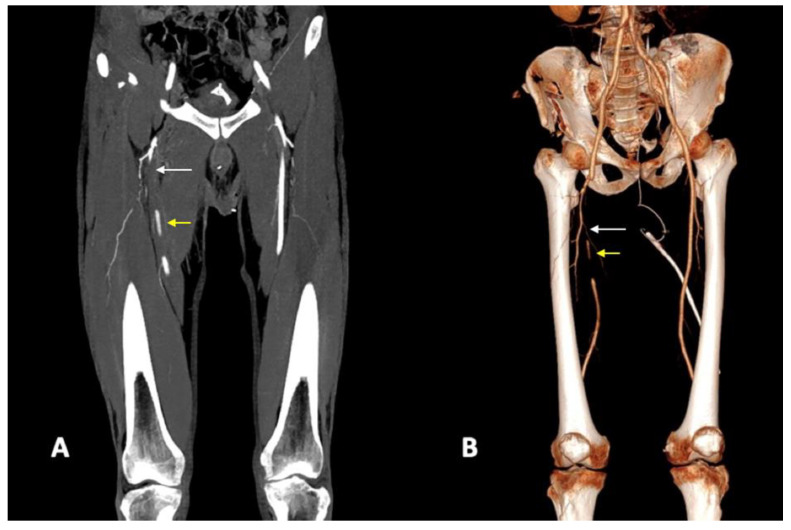
CTA, coronal planes, MIP (**A**), and 3D reconstructions (**B**). Arterial transections of proximal and medium tracts of right superficial femoral artery. In this patient, one may note both the complete loss of opacification of the proximal tract (white arrows) and the lower opacification of the downstream revascularized tract (yellow arrows) of the right superficial femoral artery, with reduced luminal caliber (narrowing).

CTA features of arterial traumas reflect the depth of mural involvement, and they are characterized by different CT findings (Figure 3) [5,65,66]:

*Arterial transection* represents the complete rupture of the vessel and determines the loss of distal opacification. Complete arterial transection may be accompanied by vessel retraction and subsequent thrombosis or a massive hematoma with active bleeding [5,8] (Figure 4). Active arterial bleeding is visualized as contrast extravasation in the arterial phase, which enlarges in the venous and delayed phases [67]. In the partial section, the arterial laceration affects the three layers of the vascular wall; without affecting the entire circumference of the vessel, the distal opacification is appreciable even if a reduced caliber and opacification of the lumen can be detected [5,55]. Partial arterial transection is more associated with massive hemorrhage than complete transection, which is more prone to arterial thrombosis [8].

*Pseudoaneurysm* is caused by focal arterial wall tear involving intimal and medial layers and represents a collection of blood contained only by the adventitia layer or surrounding tissue [5,56,59]. 

**Figure 5:**
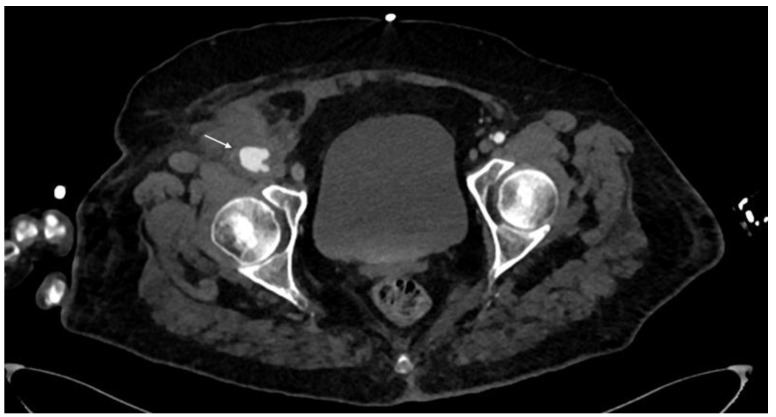
CTA, axial planes. Right common femoral artery pseudoaneurysm can be noted (arrow). It appears as an outpouching sac with a round margin in continuity with the arterial-adjacent lumen. In this case, imminent signs of rupture of the pseudoaneurysm can be seen as irregular and lobulated margins and the adjacent hematoma.

It appears as an outpouching sac with a round and smooth margin in continuity with the arterial-adjacent lumen (Figure 5). Pseudoaneurysm bleeding appears as an irregular, lobulated, perilesional contrast blush [65,68]. Pre-existing calcification or pseudoaneurysm should be differentiated from active bleeding; delayed phase acquisition can be useful because in active bleeding, the contrast extravasation dissipates along tissue planes instead of pseudoaneurysm, and calcification remains stable [60,67].

**Figure 6:**
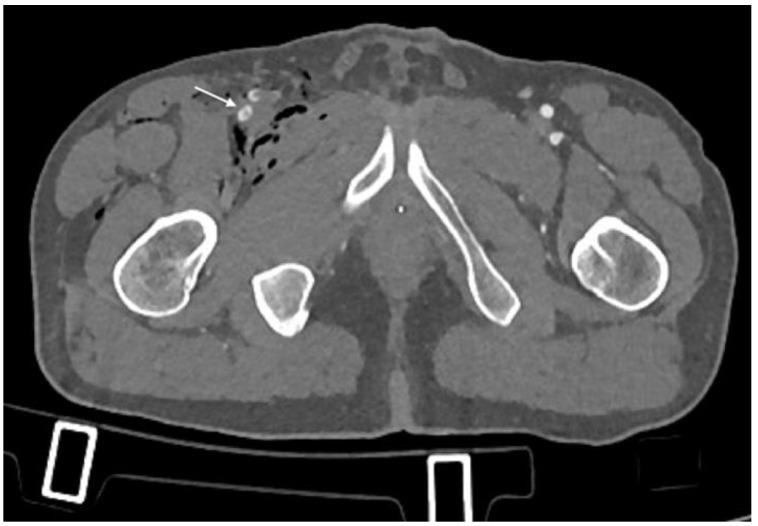
CTA, axial planes. Right deep femoral artery dissection can be seen (arrow), resulting in a linear flap within the vessel lumen.

*Dissection* is caused by an intimal tear, resulting in an intimal flap, which can float in the vessel lumen or cause occlusion [5]; at CT, it appears as a semilunar luminal deformation or eccentric stenosis or complete occlusion. Findings in dissection can be subtle, but if evident at CT, the intimal flap can be classically seen as a linear flap within the vessel lumen [5,65,69,70] (Figure 6).

In *luminal narrowing*, the vessel wall appears lobulated with eccentric narrowing; it can be the result of extrinsic compression, non-occlusive thrombus, or dissection (Figure 4).

*Vasospasm* is represented by a concentric, focal, and segmental luminal narrowing with a smooth margin, caused by the contraction of the arterial wall as a response to an injury [5]. It can be difficult to differentiate from an intimal tear and occlusion in distal small arteries [71,72]. The differential diagnosis between vasospasm and dissection often requires DSA for the proper management.

**Figure 7:**
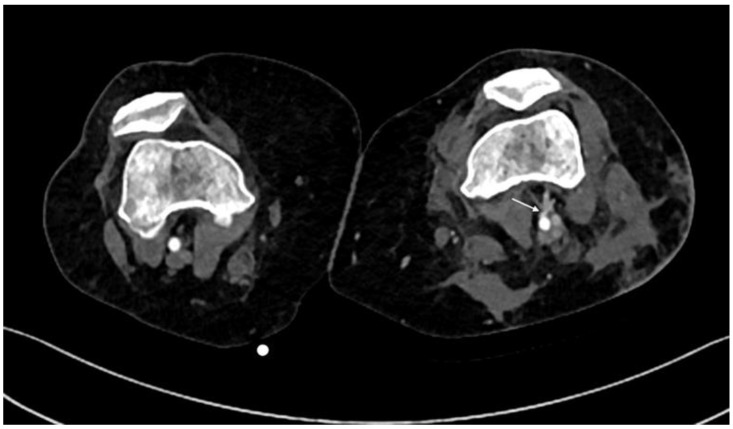
CTA, axial planes. Left popliteal arteriovenous fistula: A direct connection between the artery and the vein with early venous enhancement in the arterial phase and communicating channel with the artery can be detected (arrow).

*Arteriovenous fistulas* appear as a direct connection between arteries and veins with early venous enhancement in the arterial phase, and a communicating channel with the artery can be detected [67,68,70] (Figure 7).

### 3.3. MDCT: How to Perform and How to Report 

MDCT exam acquisition in peripheral vascular trauma can be challenging. In isolated limb trauma, CTA of lower or upper limbs can be acquired with a specific protocol, considering that optimal acquisition of upper limbs in traumatic patients can be difficult and not optimal because of the limitation to raising both arms owing to injury. Bolus tracking, fixed delays, and test injection are recommended. A fixed delay of 20–30 s in healthy patients has been proposed to adequately image both upper and lower limbs [60,66]. In polytraumatized patients, an adequate examination of the upper and lower limbs can be challenging, and 8% of extremity-trauma CTAs report nondiagnostic [66] due to early scan timing for body trauma assessment. With a second contrast arterial bolus and with the advent of a dual source, the midcalf and forearm can be reimaged with the same bolus and minimal venous opacification interference [65,73]. It is preferable to use a wide field of view that includes both limbs, which helps the radiologist to assess vascular trauma by comparing the two sides and to determine technique-equivocal findings due to distal nonenhancement for early scan timing [72]. Other factors that may negatively influence CTA diagnostic accuracy are artifacts related to beam hardening from hardware, ballistic fragments, and debris [73]. Mechanisms of trauma should be considered, especially in penetrating gunshot injuries, to adopt higher peak kilovoltage and tube current, narrow collimation, and iterative protocol to reduce artifacts [5].

CTA reporting should include a description of arterial damage with its location and length, degree of stenosis (>50% luminal caliber), and level of restitution [68]. The precise determination of the length between the transition point of a normal artery and an abnormal artery can be difficult to assess, especially in the case of a long, non-opacified segment. The accuracy in determining the extension of vascular lesions can be underestimated due to adjacent soft tissue [5,74]. In penetrating trauma, wound tracks or ballistic fragments within 5 mm of a neurovascular bundle must be considered suggestive of vascular injury [75].

Madhuripan et al. [67] proposed a systematic approach to CTA that can be useful in clinical practice. The exam should be first evaluated to assess the quality of vessel opacification. MIP (maximum intensity projection) is useful for a first and fast primary assessment of exam quality and major findings. MPR and 3D images provide a global view of findings, and if possible, comparing both limbs could help point out the lesion that should be conformed on axial images. Each vessel should be examined on axial images carefully for caliber, wall alteration, opacification, and extravasation. Meticulous assessment of major vessels run-off is relevant, and major branches must be followed along their course. Particular attention should be given to perforators in both upper and lower limbs, especially in penetrating trauma. Smaller-branch opacification of the arches of hands and feet is variable, and vascular damage should be ruled out in case of distal ischemia and no proximal vascular damage. The postprocessing workstation can be used along with optional vascular tools to aid the diagnosis. Assessment of nonvascular structures should be carried out in standard and bone windows (fractures, hematomas, soft tissue, lacerations, and foreign bodies). Incidental findings should be reported [67].

### 3.4. CTA Pitfalls

**Figure 8:**
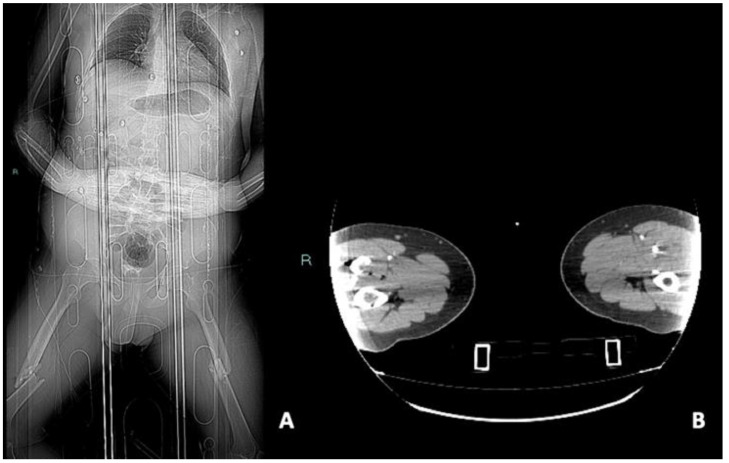
CTA scout (**A**) and arterial phase, axial planes (**B**). In this case, the correct positioning with a wide field of view was impossible, causing a nondiagnostic examination. When these conditions happen, a second limb acquisition is essential and could be performed by decentralizing the patient on the CT table and focusing the exam on the limb of interest.

Correct positioning with a wide field of view is essential but not always possible, causing a nondiagnostic examination [76] (Figure 8). A second limb acquisition could be performed by decentralizing the patient on the CT table and focusing the exam on the limb of interest. 

Poor distal opacification may occur if adequate flow is not obtained or because of delays due to cardiac output; in the latter situation, bolus tracking may be helpful [66]. Non-optimal opacification, especially of distal arteries, may be avoided with a second contrast bolus or with a second acquisition immediately after the first arterial phase. In penetrating trauma or in case of severe compressing hematomas, a delayed phase may be acquired to determine late extravasation [76].

**Figure 9:**
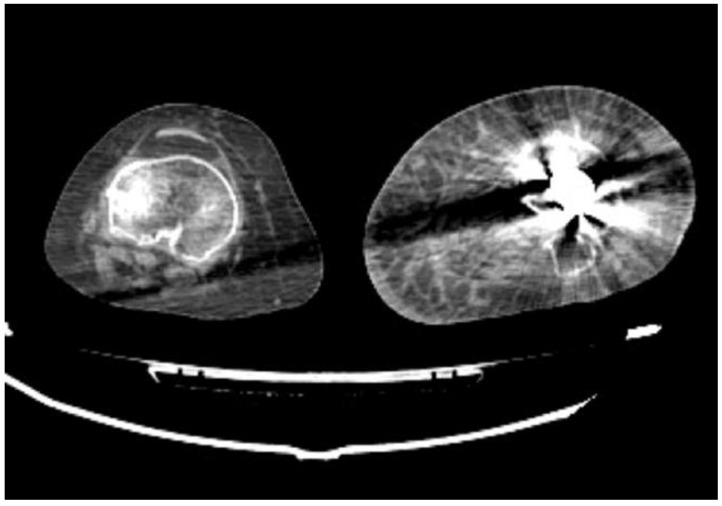
CTA, arterial phase, axial planes. In this patient, artifacts from metal arthroplasty of the left lower limb made the examination nondiagnostic. An iterative filter must be applied in order to reduce these artifacts.

Motion artifacts should be avoided when immobilizing the patients [66]. Streak artifacts from metallic fragments could lead to CT diagnostic inaccuracy (Figure 9); the iterative filter should be applied to reduce these artifacts, and distal vessels should be carefully examined [66]. In comminuted fracture, radiologists must pay attention to individuating active bleeding among bony fragments, comparing the unenhanced and arterial phases. Satisfaction errors should be avoided because 12% of patients present concomitant multiple vascular injuries [7,38,60,66,67,70,76,77,78,79].

### 3.5. CTA Timing in Peripheral Vascular Injury Assessment

An ABI of >0.9 at clinical examination generally excludes the need for additional imaging. On the other hand, patients with hard signs of PVI should be directed to the operating room, and in multiple penetrating and blunt vascular trauma, a hybrid operating room also allows the performing of angiography for diagnostic and therapeutical purposes without delay in treatment [11,12]. 

Hemodynamically unstable patients with soft signs of PVI should be directed to the operating room for resuscitation and appropriate evaluation/intervention [7]. The other category of patients that require immediate exploration without the need for imaging includes patients with peripheral vascular injury and signs of ischemia [80,81,82].

In hemodynamically stable patients with concerns of PVI, for additional evaluation (ABI and ultrasound evaluation), the presence of peripheral pulses alone cannot reliably exclude vascular injuries, and the presence of clinical signs requires further investigation [77]. Since 2012, the Eastern Association for the Surgery of Trauma (EAST) guidelines [7,79] have considered CTA the first-line modality for investigating blunt and penetrating PVIs [78] with clinical suspicion of PVI. DSA in these patients should be reserved for interventional purposes or if CTA is nondiagnostic or inconclusive due to artifacts from retained metallic objects [7,69].

## 4. Peripheral Vascular Injury Grading

Characteristically peripheral vascular injuries (PVI) are graded based on location and not on the type of lesion, according to the AAST Organ Injury Scale grading of PVI (Table 2), and they can be distinguished into occlusive or nonocclusive, depending on vascular patency [7]. Nonocclusive injuries are intimal irregularity/tear (Grade I, <25% narrowing), dissection/intramural, hematoma (Grade II, ≥25% narrowing), or partial transection with pseudoaneurysm formation (Grade III). Occlusive injuries include thrombotic occlusion (Grade IV, vessel wall is preserved) or complete transection (Grade V) [7]. The more common arteries involved are radial and ulnar arteries in the upper limb and the popliteal and superficial femoral arteries in the lower limbs [6,7]. Depending on the percentage of vessel circumference involved, the lesion should be upgraded if more than 50% of vessel circumference is involved and oppositely downgraded if less than 25% of vessel circumference is involved. 

## 5. Complications of Peripheral Vascular Injuries

Davenport et al. [15] reported the poor prognostic factors of vascular injury repair. First, a delay in treatment (>6 h) determines irreversible muscle damage [9,83]. Other negative prognostic factors that should be considered are pre-existing chronic lower-limb ischemia, initial clinical presentation with limb ischemia, injury to lower extremities, and an absent Doppler ultrasound signal at hospital admission. Factors that also negatively influence the outcome are a blunt traumatic mechanism and other associated injuries requiring immediate treatment. Pseudoaneurysms are more common in penetrating and iatrogenic trauma, and they are determined by a partial vessel disruption with bleeding in a contained intramural hematoma. Arteriovenous fistulas are more frequent in penetrating trauma, and they can develop later as a pulsatile and palpable thrill. Compartment syndrome develops when the pressure within the muscular compartment rises above 30 mmHg, and it is caused by a reperfusion injury after ischemia and is more common in young males with muscle mass [84]. Predisposing factors to compartment syndrome are crush injury, prolonged hypotension, arterial occlusion or a combined arterial and venous injury, and vein ligation. It more commonly occurs because of tibial shaft fractures or distal radius fractures [84], and CT imaging is not advocated for the diagnosis of compartment syndrome, although at CT, muscle enlargement with focal or geographic areas of hypoattenuation may indicate rhabdomyolysis, and intramuscular collection with peripheral rim enhancement may indicate signs of myonecrosis [85]. Early fasciotomies need to be considered, and high suspicion should be carried out in these patients. Unluckily, amputation can represent the first line of treatment in case of severe soft-tissue damage, irreversible ischemia, and neurological damage. Amputation may also be determined by a delay in the diagnosis of popliteal artery and crural vessel damages or if there have been delays in resuscitation.

## 6. Conclusions

CTA of peripheral vascular trauma requires knowledge of vessel anatomy and a deep understanding of trauma mechanisms in order to properly identify lesions.

## Figures and Tables

**Table 1:** CTA protocol for upper- and lower-extremity injury.

Parameter	Details
Position	Supine feet first
Scan range	Caudal-cranial direction from the inferior aspect of the aortic arch to the tips of the fingers (for upper-limbs trauma) Cranio-caudal direction when the upper-limbs are placed above the head or for lower-limbs trauma
Acquisitions	Unenhanced scan suggested Arterial phase (bolus tracking and minimum delay) Venous phase (60–70 s after the contrast agent injection) Late phase (180 s after the contrast agent injection)
Trigger	Bolus tracking; trigger at the aortic arch for the upper-limb examinations or in the whole-body CT examination, and on the abdominal aorta in case of lower-limb examinations
kVp	90-130
mAs	Auto-modulation
Slice thickness	≤1.5 mm
Iodine Delivery Rate (IDR)	1.4–1.8 gI/s
Amount	(IDR/iodine concentration) x injection duration

**Table 2:** AAST Organ Injury Scale Grading of peripheral vascular injury [80].

Grade	Injury
I	Digital artery/vein, palmar artery/vein, deep palmar artery/vein, dorsal pedis artery, planter artery/vein, non-named arterial/venous branches.
II	Basilic/cephalic vein, saphenous vein, radial artery, ulnar artery.
III	Axillary vein, superficial/deep femoral vein, popliteal vein, brachial artery, anterior tibial artery, posterior tibial artery, peroneal artery, tibio-peroneal trunk.
IV	Superficial/deep femoral artery, popliteal artery.
V	Axillary artery, common femoral artery.

## Data Availability

Not applicable.
